# High-Precision, *In Vitro* Validation of the Sequestration Mechanism for Generating Ultrasensitive Dose-Response Curves in Regulatory Networks

**DOI:** 10.1371/journal.pcbi.1002171

**Published:** 2011-10-06

**Authors:** Francesco Ricci, Alexis Vallée-Bélisle, Kevin W. Plaxco

**Affiliations:** 1 Department of Chemistry and Biochemistry, University of California, Santa Barbara, California, United States of America; 2 Dipartimento di Scienze e Tecnologie Chimiche, University of Rome, Tor Vergata, Rome, Italy; 3 Consorzio Interuniversitario Biostrutture e Biosistemi “INBB”, Rome, Italy; 4 Interdepartmental Program in Biomolecular Science and Engineering, University of California, Santa Barbara, California, United States of America; Chinese Academy of Sciences, China

## Abstract

Our ability to recreate complex biochemical mechanisms in designed, artificial systems provides a stringent test of our understanding of these mechanisms and opens the door to their exploitation in artificial biotechnologies. Motivated by this philosophy, here we have recapitulated *in vitro* the “target sequestration” mechanism used by nature to improve the sensitivity (the steepness of the input/output curve) of many regulatory cascades. Specifically, we have employed molecular beacons, a commonly employed optical DNA sensor, to recreate the sequestration mechanism and performed an exhaustive, quantitative study of its key determinants (*e.g.,* the relative concentrations and affinities of probe and depletant). We show that, using sequestration, we can narrow the pseudo-linear range of a traditional molecular beacon from 81-fold (*i.e.,* the transition from 10% to 90% target occupancy spans an 81-fold change in target concentration) to just 1.5-fold. This narrowing of the dynamic range improves the sensitivity of molecular beacons to that equivalent of an oligomeric, allosteric receptor with a Hill coefficient greater than 9. Following this we have adapted the sequestration mechanism to steepen the binding-site occupancy curve of a common transcription factor by an order of magnitude over the sensitivity observed in the absence of sequestration. Given the success with which the sequestration mechanism has been employed by nature, we believe that this strategy could dramatically improve the performance of synthetic biological systems and artificial biosensors.

## Author Summary

Here we recreate *in vitro* the sequestration mechanism thought to underlie the extraordinary sensitivity (the steepness of the input/output function) of a number of genetic networks. We do so first using fluorescent molecular beacons, a well-established, DNA-based biosensor architecture, as our model system. The experimental parameters that define this *in vitro* model can be controlled with great precision, allowing us to dissect and test a quantitative model of sequestration in unprecedented detail. Following on this we employ the sequestration mechanism to steepen the binding-site occupancy curve of a common transcription factor by an order of magnitude over the sensitivity observed in the absence of sequestration. Our study thus highlights the versatility with which this approach can be used to improve the performance of both synthetic biological systems and artificial biosensors.

## Introduction

In order to test the extent to which we understand complex biochemical systems -and to improve our ability to exploit them in man-made technologies (e.g., synthetic biology; biosensors)- it is important to reconstruct these processes in the laboratory. Illustrative examples of this include recent demonstrations of synthetic genetic networks in which genetic elements are “mixed and matched” in order to create artificial bistable “toggle switches,” genetic oscillators and other complex, non-linear input/output behaviors (*e.g.,* [1]-[5]). Other examples include the recent *de novo* design of proteins, including enzymes, unrelated to any naturally occurring sequences (*e.g.,* [6]-[9]) and the artificial selection of new proteins [10]-[11]. And while these studies do not (and cannot) prove that our knowledge of, for example, genetic regulatory networks and protein folding and evolution is exhaustively complete, they nevertheless suggest that our understanding of these naturally occurring systems is sufficient to enable the design of similarly complex, artificial systems [12].

**Figure 1:**
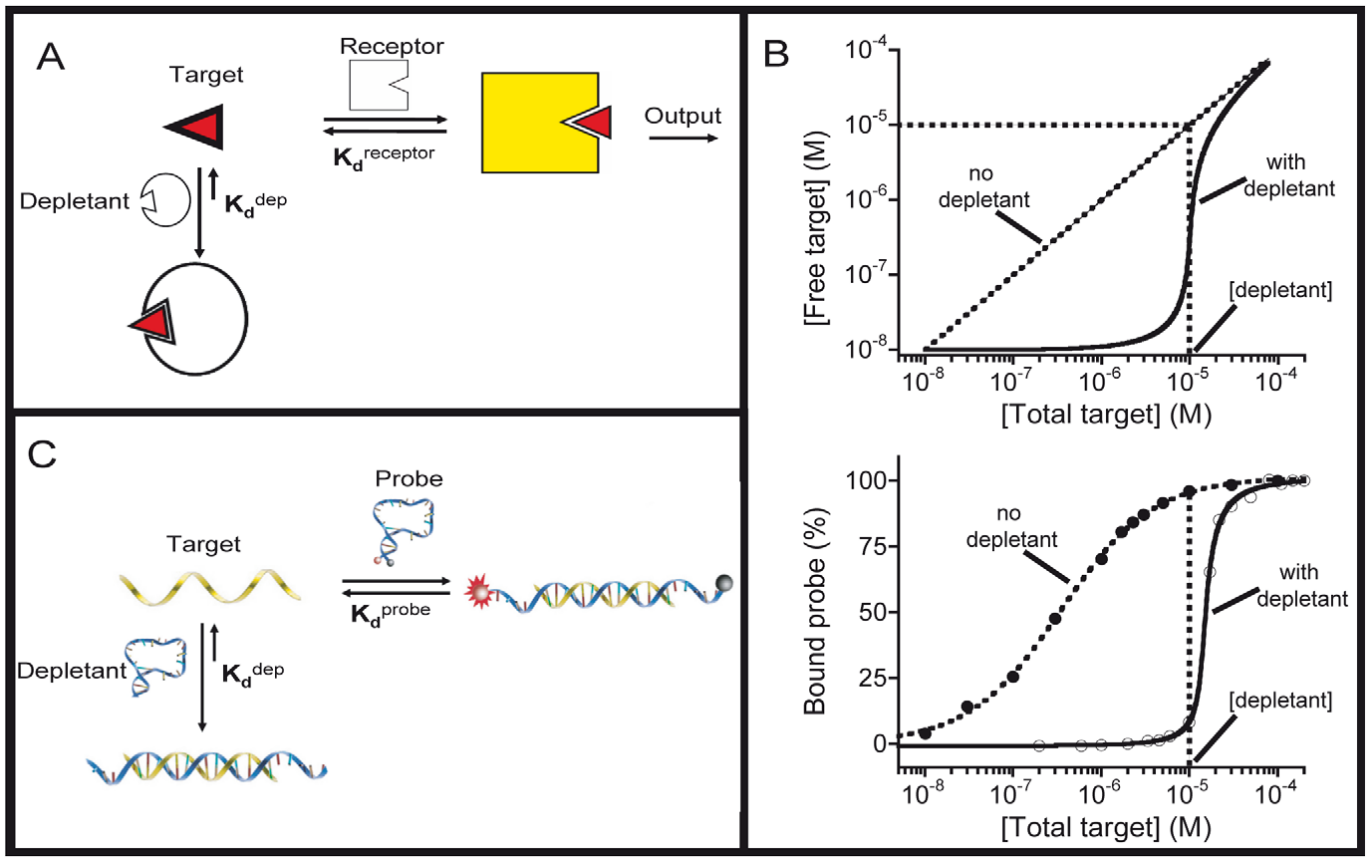
*In vitro* recreation of the sequestration mechanism. Here we recapitulate *in vitro* the sequestration mechanism employed by nature to produce ultrasensitive outputs (steep input/output functions) in a number genetic regulatory networks. (**a**) In the sequestration mechanism low concentrations of a target molecule are “depleted” by binding to a high affinity non-signaling receptor that acts as a “sink” (the depletant). (**b**) *Top,* when the total target concentration surpasses the concentration of the depletant (the sink is saturated), a threshold response is achieved in which, upon the addition of any further target, the relative concentration of free target rises rapidly. *Bottom*, this threshold effect generates a “*pseudo*-cooperative” dose-response curve in which probe occupancy, and thus the output signal, arises much more rapidly than would occur in the absence of a depletant. (**c**) As a test bed to recapitulate and exploit this mechanism we have employed DNA molecular beacons, widely used probes for the detection of specific oligonucleotide sequences [29]. Consisting of a stem-loop DNA modified with a fluorophore/quencher pair the affinities of molecular beacons can be tuned by changing the stability of their stems [33], thus rendering it easy to generate depletant (unlabeled molecular beacons) with almost any arbitrary affinity.

Motivated by the above philosophy, here we recreate *in vitro* the “sequestration” mechanism thought to underlie the extraordinary sensitivity (the steepness of the input/output function) of a number of genetic networks (*e.g.,* [5], [13]-[18]). In the sequestration mechanism, low concentrations of a given target molecule are sequestered by binding to a high affinity (low dissociation constant) receptor that acts as a “depletant,” which serves as a “sink” that prevents the accumulation of free target without generating an output signal (Figure 1a). When the total target concentration surpasses the concentration of the depletant (saturating the sink), a threshold response is achieved in which the addition of any further target produces a large rise in the relative concentration of free target (Figure 1b, top). The rapidly rising concentration of free target then binds to –and thus activates– a second, lower affinity (higher dissociation constant) receptor (or “probe”) that, unlike the depletant, generates an output signal. This threshold effect generates a “pseudo-cooperative” dose-response curve, which is much more sensitive (much steeper) than the hyperbolic “Langmuir isotherm” produced by simple, single site binding (Figure 1b, Bottom) [17]-[19].

(At this juncture we must note an important semantic distinction. The *sensitivity* of biological systems, such as metabolic networks or signal transduction pathways, is defined as the ratio of the relative change in output to the relative change in input [15], [19]. The term *ultrasensitivity* thus describes systems for which the upstroke of the input/ouput function is steeper than the simple, hyperbolic curve obtained for single site binding, such as is observed for “classic” Michaelis-Menten enzymes [15], [18]. This definition of sensitivity is distinct from “*analytical sensitivity*,” which represents the smallest input (rather than the smallest *change* in input) that the method is capable of resolving above the noise floor. Indeed, as we show here, ultrasensitive behaviour is often produced at the cost of a reduced analytical sensitivity as a steeper input/output function is often achieved at the cost of increasing the smallest input signal that can be robustly detected. In this paper we use only the former, steep-input/output-function definition of the terms “sensitivity” and “ultrasensitivity.”).

Sequestration is thought to underlie the ultrasensitive responses of many cellular processes. An example is the depletion of specific messenger RNA by the binding of small regulatory RNA, which generates ultrasensitive thresholds leading, in turn, to the highly sensitive regulation of gene expression [20]-[24]. The binding of many transcription factors is likewise thought to be rendered ultrasensitive via a sequestration mechanism in which high affinity “decoy” binding sites scattered across the genome (or inhibitory proteins that compete for the transcription factor [25]-[26] act as depletants leading to a steep, effectively “all-or-none” transcriptional response [13], [27]-[28]. As a test of this hypothesis, Buchler and co-workers have recently recreated the sequestration mechanism *in vivo* in a synthetic genetic circuit that converts a graded transcriptional response into an ultrasensitive response via the addition of a depletant [16]. Here we build on their study by recreating the sequestration mechanism *in vitro* using molecular beacons, a well-established biosensor architecture, as our model system. The experimental parameters that define this *in vitro* model can be controlled with great precision, providing a means to dissect and test a quantitative model of sequestration in unprecedented detail. Following this we have adapted the sequestration mechanism to steepen the concentration-occupancy relationship for the binding of a common transcription factor by an order of magnitude over the sensitivity observed in the absence of sequestration.

## Results

Molecular beacons, synthetic biomolecular switches developed by Kramer and coworkers for the detection of specific DNA or RNA sequences [29], are now widely used in the diagnosis of genetic and infectious diseases [30]-[32]. Consisting of a stem-loop DNA modified with a fluorophore/quencher pair, molecular beacons are quantitatively described by a simple three-state population-shift model in which the equilibrium between a non-binding, non-signaling state and the binding-competent, signaling state is pushed towards the latter upon target binding [33]. This linkage between a conformational equilibrium and target binding allows us to rationally tune the affinity of molecular beacons –without affecting their specificity– by altering the stability of the stem. Indeed, using this approach we have previously shown that it is possible to tune the affinity of molecular beacons across more than 4 orders of magnitude [33]. For the studies reported here we have used a set of six molecular beacons sharing a common recognition element but spanning this same 10,000-fold range of target affinities (Table 1). The input/output function of each of these six molecular beacons is well described by the hyperbolic curve expected for single site binding,
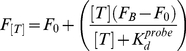
(1)


in which *F_[T]_* is the fluorescence output as a function of target concentration, [*T*], *F_0_* and *F_B_* are the fluorescence of the unbound and bound states respectively, and *K_d_^probe^* is the dissociation constant of the probe/target duplex.

**Table 1:** The affinities of the stem-loop constructs employed here.

	*K_d_^probe^* (nM)^a^	*K_d_^dep^* (nM)^b^
0GC	13 (±2)	5.2
1GC	42 (±4)	6.6
2GC	310 (±20)	11.3
3GC	2,600 (±40)	36.9
4GC	not used	225.8
5GC	not used	3,647

^**a**^FAM/BHQ-1 modified molecular beacons are ∼8 kJ more stable than their unlabeled stem-loop counterparts due to interactions between the dye and the quencher [47]; their affinities for a 13-base sequence targeting the loop have been determined as reported elsewhere [33]. **^b^** *K_d_* of the unlabeled depletant constructs were calculated using their simulated switching equilibrium and the expected *K_d_* of the open form of the molecular beacon [33].

**Figure 2:**
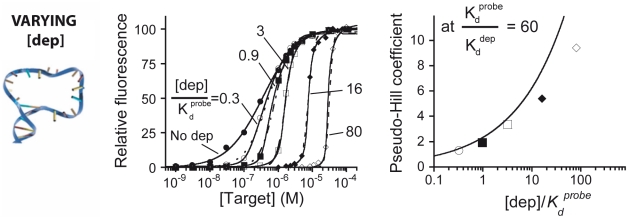
Ultrasensitivity is a strong function of the ratio [dep]/*K_d_^probe^*. Using molecular beacons, we have recapitulated the sequestration mechanism *in vitro* and, in so doing, have vastly increased the sensitivity of this commonly employed biosensor. **(Left)** We find that ultrasensitivity is a strong function of the ratio [dep]/*K_d_^prob^* ^e^, which measures the extent to which the concentration of the depletant rises above the affinity of the probe. (Here we are using a depletant/probe pair for which *K_d_^probe^/K_d_^dep^*  =  60). To quantify the sensitivity of these dose response curves we have fitted them to the Hill equation (dotted lines) to define pseudo-Hill coefficients. (**Right**) This pseudo-Hill coefficient increases monotonically as the [dep]/*K_d_^prob^* ^e^ ratio increases, reaching 9.4 at the highest ratios we have investigated. It is important to note, however, that although the Hill coefficient provides an easy way to compare sensitivity across different systems, the Hill equation is not a correct physical description of our system. Instead, the behavior of our system is described by the sequestration model as expressed in equation 2 (see text; see also Buchler *et al.,* 2009). Using equation 2 and the previously determined dissociation constants of our probe and depletant [33], we can model the sensitivity of this system quantitatively (solid lines, left panel) without the use of *any* floating parameters. The theoretically modeled pseudo-Hill coefficient likewise describes our data quite well (solid line, right panel), deviating only slightly at our highest ratios we have investigated.

We introduce sequestration into molecular beacons by combining a relatively low affinity signaling probe (*i.e.,* fluorophore/quencher labeled) with an excess of a higher affinity (but unlabeled and thus non-signaling) stem-loop that serves as the depletant (dep) (Figure 1c). Doing so, we convert the hyperbolic binding curve associated with a traditional molecular beacon (Eq. 1) into a much steeper, ultrasensitive response (Figure 2).

A physically reasonable description for the proposed sequestration mechanism is easily derived from the above hyperbolic binding curve by replacing [T] with the effective concentration of free (unbound, un-sequestered) target. As per Buchler and Louis [15], this concentration goes with: 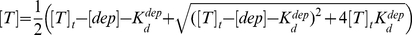
(2)


where [*T*]*_t_* is the total amount of target added and *K*_d_^dep^ is the dissociation constant of the depletant/target complex. By combining Eq. 1 and Eq. 2 we see that the ratio of the depletant concentration to the probe affinity ([dep]/*K_d_^probe^)* is a crucial determinant of ultrasensitivity. To validate this we employed the relatively low affinity molecular beacon 2GC_probe_ (*K_d_^probe^*  =  310 nM; table 1) as our probe and the higher affinity unlabeled molecular beacon 0GC_dep_ (*K_d_^dep^*  =  5.2 nM) as our depletant. When we do so we observe ultrasensitivity as soon as the depletant concentration rises above the probe dissociation constant (*i.e.,* as [dep]/*K_d_^probe^* increases above unity; Figure 2, left). With further increases in depletant concentration the steepness of the dose-response curve increases monotonically to the highest [dep]/*K_d_^probe^* ratios we have investigated. Moreover, by applying equations 1 and 2 to these data we see that the sequestration model fits these ultrasensitive responses quantitatively (*R^2^* ≥ 0.998) *without the use of any fitted parameters* (solid lines, Figure 2). That is, we can quantitatively fit our observations with this model using parameters values –the affinities and fluorescence signals of the two molecular beacons- determined independently in previous studies [33].

The Hill coefficient is commonly employed to describe ultrasensitive systems in biochemistry [34]. And while it is not a physically correct description of sequestration (as it was originally derived to describe allosteric cooperativity), we find that the *pseudo*-Hill coefficient we obtain by fitting the Hill equation to our data provides a convenient way of comparing the ultrasensitive behavior generated by the sequestration mechanism. As expected, we observe a pseudo-Hill coefficient near unity (0.90±0.02) for a binding curve obtained in the absence of depletant (dotted lines in Figure 2, left). Upon the addition of depletant this value climbs, reaching 1.3 at a [dep]/*K_d_^probe^* ratio of 0.3 before ultimately reaching a value of 9.4 at a ratio of 80, the highest [dep]/*K_d_^probe^* ratio we have investigated (Figure 2, right). That is, sequestration ultimately compresses the normally 81-fold dynamic range of a molecular beacon (*i.e.,* the transition from 10% to 90% target occupancy spans an 81-fold change in target concentration; see ref. 17) into a 1.5-fold dynamic range, significantly increasing the steepness of the input/output function of the molecular beacon and, in turn, improving its ability to detect small changes in relative target concentration.

**Figure 3:**
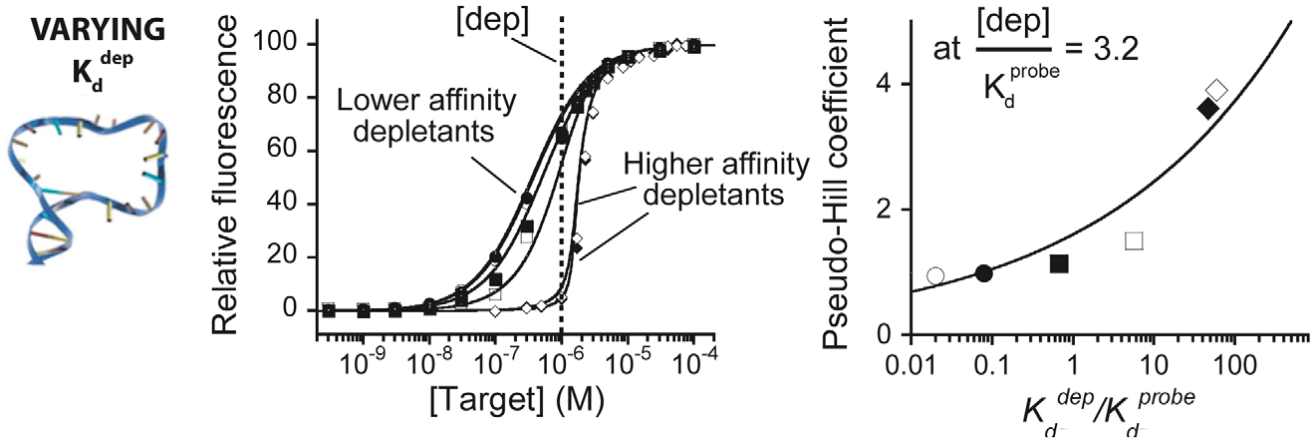
The relative affinities of the depletant and probe play a crucial role in generating ultrasensitivity. For example, if, as is true here, [dep]/*K_d_^pro^* ^be^  =  3.2, we only achieve significant sensitivity when the affinity of the depletant is at least 10 times greater than that of the probe. (**Left**) To demonstrate this we present here binding curves obtained with a medium affinity molecular beacon (2GC_probe_, *K_d_^probe^*  =  310 nM) in the presence of depletants ranging in affinity from 5.2 nM to 3 µM (the depletants are, from right to left: 0GC_dep_, 1GC_dep_, 2GC_dep_, 3GC_dep_, 4GC_dep_ and 5GC_dep_). Higher affinity depletants (0GC_dep_, 1GC_dep_), those with dissociation constants at least 10 times lower than that of the probe, produce clear, ultrasensitive responses. In contrast, depletants with affinities similar to (2GC_dep_, 3GC_dep_) or poorer than (4GC_dep_ and 5GC_dep_) that of the probe produce little improvement in sensitivity. (**Right**) As demonstrated above the experimentally observed pseudo-Hill coefficients compare well with the theoretically modeled values (solid line). Again, the solid lines in these panels are not fits. Rather they are estimates taken directly from equation 2 (and using the known dissociation constants of the relevant probes and depletants) without the use of *any* fitted parameters.

Our *in vitro* model also provides an opportunity to explore, for the first time, the extent to which sequestration-derived ultrasensitivity depends on other parameters, including, for example, the relative affinities of the depletant and the probe (*i.e., K_d_^probe^/K_d_^dep^*). To do so, we varied the depletants affinity (using *K_d_* ranging from 5.2 nM to 3 µM) at a constant [dep]/*K_d_^prob^* ^e^ ratio of 3.2 (*K_d_^probe^*  =  310 nM –molecular beacon 2GC- with and a [dep] of 1 µM). As expected we find that, while high affinity depletants (*i.e.,* 0GC_dep_, 1GC_dep_) produce clear ultrasensitive responses (pseudo-Hill coefficients of 3.9 and 3.6 respectively), depletants with affinities similar to (*i.e.,* 2GC_dep_, 3GC_dep_) or poorer than (*i.e.,* 4GC_dep_ and 5GC_dep_) those of the probe produce only minor improvements in sensitivity (pseudo-Hill coefficients <1.3) (Figure 3, left). Again, all of the data so obtained are well modeled by Eq. 1 and 2 without the use of any fitted parameters (*i.e.,* by fixing all parameters to the values obtained from independent experimental conditions), providing another high precision test of the quantitative sequestration model (Figure 3, right).

**Figure 4:**
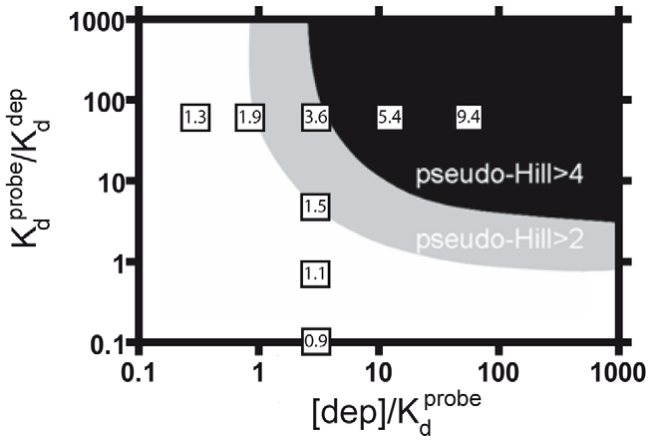
Simulation of the sequestration mechanism. A simulation of equations 1 and 2 illustrates the range of [dep]/*K_d_^probe^* and *K_d_^probe^/K_d_^dep^* over which pseudo-Hill coefficients above 2 (gray area) and above 4 (dark area) are obtained. Shown in boxes are some of the experimental pseudo-Hill coefficients we have observed (the horizontal and vertical lines of data were taken from figures 2 and 3 respectively).

While the two ratios described above, [dep]/*K_d_^probe^* and *K_d_^probe^/K_d_^dep^*, play crucial roles in generating ultrasensitivity they do not work independently of one another. For example, if [dep]/*K_d_^probe^* falls well below unity we will not obtain ultrasensitivity no matter how high the *K_d_^probe^/K_d_^dep^* ratio climbs. This occurs because, when the probe dissociation constant is significantly higher than the concentration of the depletant, the free target concentration at the “threshold” is too low to saturate the probe, leading to a more-or-less hyperbolic response approximating that seen in the absence of depletant (Figure S1). To better understand the interplay between these two ratios (*i.e.,* to illustrate the parameter space over which ultraensitive behavior is obtained) we can plot the pseudo-Hill coefficient as a function of [dep]/*K_d_^probe^* and *K_d_^probe^/K_d_^dep^* (Figure 4). Doing so we find that, when [dep]/*K_d_^probe^* falls below 0.91 it is not possible to achieve ultrasensitivity with any reasonable value of *K_d_^probe^/K_d_^dep^*. Similarly, if *K_d_^probe^/K_d_^dep^* falls below 0.94 we do not generate a pseudo-Hill coefficient above 2 even at the highest depletant concentration we have employed.

Finally, the ease with which we can manipulate our *in vitro* system renders it possible to also characterize the effects of varying *K_d_^prob^* ^e^ at a constant depletant concentration. To do so, we increased the length of our target sequence, which lowers the dissociation constants of the probe and depletant for the target by the same extent. Using targets ranging from 13 to 17 nucleotides (and thus increasing the [dep]/*K_d_^probe^* ratio from 0.3 to more than 100), we observe the expected monotonic increase in sensitivity (Figure S2). Moreover, these data too, fit equations 1 and 2 quantitatively (*R^2^>*0.995) without the use of any adjustable parameters.

**Figure 5:**
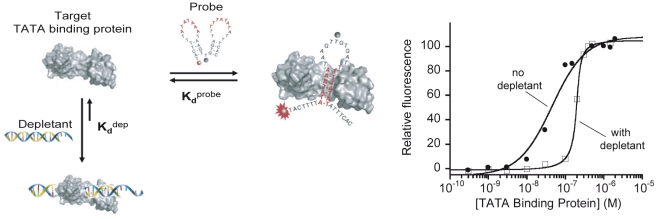
Using the sequestration mechanism to steepen the binding-site occupancy curve of a transcription factor. We have used the sequestration mechanism to steepen the concentration/occupancy curve of a common transcription factor by an order of magnitude over the sensitivity observed in the absence of sequestration. (**left**) As our read-out mechanism we have employed transcription factor beacons, a recently developed, high-precision reporter of transcription factor binding site occupancy analogous to molecular beacons [36] (**right**). Using a transcription factor beacon directed against the target TATA binding protein as our probe and a 10-fold excess of a simple, higher affinity, double-stranded hairpin DNA as our depletant, we achieve an ultrasensitive dose-response curve with a pseudo-Hill coefficient of 4.3. This compresses the normal 81-fold dynamic range over which this binding site is occupied (the pseudolinear range between 10% and 90% of target occupancy) to only 4-fold, leading to a much more sensitive concentration-occupancy curve.

In the above studies molecular beacons served as a convenient, synthetic toolkit to quantitatively and precisely test the sequestration model. Obviously, however, molecular beacons are not themselves of any specific biological relevance. We have thus also developed an *in vitro* system to test the extent to which sequestration can cause pseudo-cooperative, highly sensitive changes in the concentration of free transcription factor, thus increasing the sensitivity with which a transcription factor-binding site is occupied. A difficulty in demonstrating this with precision is that traditional methods for quantifying the occupancy of a transcription-factor binding site, including gene activation, gel shift assays and ELISAs, provide only relatively “low resolution” measurements of site occupancy. As our read-out we have thus instead employed a recently developed, highly precise optical reporter for transcription factor binding activity termed “transcription factor beacon” (Figure 5, left) [35]. Specifically, in order to detect the binding of our transcription factor, TATA binding protein, we have used a transcription factor switch that exhibits a 45 nM dissociation constant for this target, reporting its binding via a large change in fluorescence output. As our depletant we have employed a hairpin DNA that contains TATA binding protein’s double stranded recognition sequence but that lacks a fluorophore/quencher reporting pair. Unlike the transcription factor switch, the hairpin does not undergo any binding-induced conformational change and thus its affinity for TATA binding protein is, as required by the sequestration mechanism, significantly greater than that of the reporting probe. Using a 1∶10 mixture of this probe/depletant pair we achieve a pseudo-Hill coefficient of 4.3, compressing the normally 81-fold psuedolinear range of the occupancy of this transcription factor binding site to a mere 4-fold and thus significantly increasing the sensitivity with which it is occupied (Figure 5, right).

## Discussion

Using molecular beacons and transcription-factor binding as model systems we have recreated, *in vitro,* the sequestration mechanism that Nature employs to generate ultrasensitive behavior in many genetic networks. Doing so we have demonstrated that the simple, quantitative model proposed by Buchler and Louis accurately and precisely predicts the relationships between ultrasensitivity and the concentrations and affinities of the depletant and probe. We have also demonstrated, more generally, the utility of employing DNA-based *in vitro* models in the high precision testing and dissection of biologically important regulatory mechanisms.

As noted above, Buchler and co-workers [15], [16] were among the first to test the sequestration mechanism using a synthetic genetic circuit *in vivo* that they converted from a graded transcriptional response into an ultrasensitive output via the addition of a depletant [16]. Their work confirmed earlier suggestions that sequestration could underlie bistable or oscillatory circuits in natural regulatory systems. It also highlighted the important determinants of the sequestration mechanism. Due to the intrinsic complexity of *in vivo* systems, however, it proved difficult to use this model system to test all the determinants of sequestration with high precision. Buchler and co-worker, for example, were unable to evaluate the range of *K_d_^probe^/K_d_^dep^* over which various degree of ultrasensitive behavior could be observed. Here, in contrast, we have employed molecular beacons, a well-defined, easily controllable, *in vitro* system, as a tool to dissect the sequestration mechanism [15], [16] in more detail and with greater precision than has hitherto proven possible.

While the impressive specificity, affinity and versatility of biomolecular recognition have motivated decades of research in the development of sensors and other biotechnologies based on this effect [36], the hyperbolic –and thus not particularly sensitive– concentration/occupancy curves characteristic of single site binding limits their precision. This, in turn, limits the utility of these biotechnologies in many applications. Given this we believe that the use of sequestration *in vitro* may be of use in increasing the sensitivity of synthetic biosystems, such as biosensors, *in vitro*. Specifically, we have shown that it possible to narrow the 81-fold pseudo-linear dynamic range of the well-known molecular beacon platform by almost 2 orders of magnitude and of a transcription factor switch sensor by a factor of 20. The modified sensors so produced exhibit ultrasensitive responses equivalent to Hill coefficients of greater than 9 and greater than 4 respectively, converting them into high precision analytical approaches. Given that sequestration requires only the availability of depletants that bind the target in question with greater affinity than that of the signal-generating probe, we anticipate that the mechanism can be adapted to many other biotechnologies, an argument bolstered by the frequency with which this mechanism is employed in the cell [15], [27], [28]. Moreover, the sequestration mechanism appears more straightforward to implement than the other mechanisms Nature has employed to achieve improved sensitivity. It appears far easier to implement, for example, than positive allosteric cooperativity as the later would involve the design of probes containing two or more precisely interacting sites for target binding [37]-[38]. Despite these advantages, the sequestration strategy is not without a limitation: the generation of ultrasensitive response is achieved at the cost of a reduced affinity, which shifts the minimum target concentration producing a detectable signal (the detection limit) towards higher concentrations.

The extremely steep input/output functions demonstrated here would appear to open the door to new applications across biosensors and synthetic biology. Perhaps the most obvious application would be in the monitoring of, for example, drugs with such narrow therapeutic windows that only high precision measurements of their concentration achieve clinical relevance. More speculatively, the availability of sensors that, in contrast to the graded (analog) outputs of most biosensors, produce an effectively “all-or-none” (digital) response may be useful in the development of molecular logic gates [39]-[43]. These, in turn, may enable the development of molecular-scale computers and “autonomously regulated” chemical systems, ideas that have attracted significant recent interest [44], [45].

## Materials and Methods

### Fluorescent DNA molecular beacons

The following HPLC-purified constructs were purchased from Sigma-Genosys and used as received (the bases in italic are those constituting the stem):

**1GC_probe_**: 5’-(FAM)-A-*CTATT-*GATCGGCGTTTTA-*AATAG*-G -(BHQ)-3’

**2GC_probe_**: 5’-(FAM)-A-*CTCTT*-GATCGGCGTTTTA-*AAGAG*-G -(BHQ)-3’

**3GC_probe_**: 5’-(FAM)-A-*CTCTC*-GATCGGCGTTTTA-*GAGAG*-G -(BHQ)-3’

where FAM and BHQ represent 6-carboxyfluorescein and Black Hole Quencher respectively.

### Unlabeled molecular beacons and targets

The following HPLC-purified, un-modified depletants and targets were purchased from Sigma-Genosys and were used as received (the bases in italic are those constituting the stem):

**0GC_dep_**: 5’-A-*TTATT* -GATCGGCGTTTTA-*AATAA*-G -3’

**1GC_dep_**: 5’-A-*CTATT* -GATCGGCGTTTTA-*AATAG*-G -3’

**2GC_dep_**: 5’-A-*CTCTT* -GATCGGCGTTTTA-*AAGAG*-G -3’

**3GC_dep_**: 5’-A-*CTCTC*-GATCGGCGTTTTA-*GAGAG*-G -3’

**4GC_dep_**: 5’-A-*CTCGC*-GATCGGCGTTTTA-*GCGAG*-G -3’

**5GC_dep_**: 5’-A-*CGCGC*-GATCGGCGTTTTA-*GCGCG*-G -3’

**13-base target:** 5’-TAAAACGCCGATC-3’

**15-base** **target:** 5’-TTAAAACGCCGATCA-3’

**17-base** **target:** 5’-TTTAAAACGCCGATCAA-3’

### Transcription factor switch

HPLC purified DNA modified with FAM and internal BHQ-1 inserted on a thymine residue was purchased from Biosearch Technologies (Novato, CA) and has the following sequence:

**TF_probe_**: 5’-(FAM)-TACTT TTATATAAAT AAGT T(BHQ) GTGA TTTTTATATATT TCAC -3’

### Transcription factor depletant

The following HPLC-purified, un-modified depletant was purchased from Sigma-Genosys and was used as received:

**TF_De_**_p_: 5’-CGTATATAAAGG TTTTTTT CCTTTATATACG -3’

### TATA binding protein

This protein was expressed recombinantly, purified, and characterized as previously described [46].

### Fluorescent measurements

All fluorescent experiments were conducted at pH 7.0 in 50 mM sodium phosphate buffer, 150 mM NaCl, at 45 °C. For all experiments with TATA binding protein, the buffer was supplemented with 5 mM MgCl_2_, as it is essential for efficient DNA binding and the measurement were conducted at 25 °C. Equilibrium fluorescence measurements were obtained using a Cary Eclipse Fluorimeter with excitation at 480 (± 5) nm and acquisition at 517 (± 5) nm. Binding curves were obtained using solutions of 10 nM of labeled molecular beacons (or TF switch) and varying concentrations of unlabeled beacons (or DNA binding protein recognition sequence) as depletant and by sequentially increasing the target concentrations *via* the addition of small volumes of solution with increasing concentrations of target. Dissociation constants of the labeled beacons were obtained from the literature [33].

## Supporting Information

Figure S1 The concentration of the depletant and the affinities of the probe and depletant sometimes interact in complex ways during the generation of ultrasensitivity. Here we demonstrate this by fixing the concentration of the depletant (0GC_dep_) at 300 nM and employing probes of differing affinities. Despite the high affinities ratio (*K_d_^probe^/K_d_^dep^*  =  500), we do not observe ultrasensitivity when the probe dissociation constant is higher than the concentration of the depletant (3GC_probe_; *K_d_^probe^*  =  2.6 µM, [dep]/*K_d_^prob^* ^e^  =  0.11), because the free target concentration at the “threshold” is too low to saturate the probe. In contrast, we achieve greater sensitivity when the dissociation constant of the probe is close to (2GC_probe_; *K_d_^probe^*  =  310 nM, [dep]/*K_d_^pro^* ^be^  =  0.97) or lower (1GC_probe_; *K_d_^probe^*  =  42 nM, [dep]/*K_d_^probe^*  =  7.1) than the depletant concentration. The solid lines are estimates taken directly from equation 2 (and using the known dissociation constants of the relevant probes and depletants) without the use of any fitted parameters.
(TIF)

Figure S2 We have also characterized the effects of varying *K_d_^prob^* ^e^ at a constant depletant concentration. We have done this by lengthening the target, which simultaneously improves its affinity for the depletant and for the probe -here 2GC- and keeping the concentration of depletant (here 0GC) constant (i.e. 100 nM). Using targets ranging from 13 to 17 nucleotides (producing lower depletant dissociation constants) we observe a monotonic increase in the pseudo-Hill coefficient from 1.3 to 4.0. This steep, ultrasensitive response is achieved despite the low, 10-fold [depletant]/[probe] ratio employed here, which usually renders the generation of ultrasensitivity more difficult (i.e. [probe]  =  10nM). The solid lines are simulations taken directly from equation 2 without the use of any fitted parameters.
(TIF)
