# Supplementary figures and images for: High-Precision, In Vitro Validation of the Sequestration Mechanism for Generating Ultrasensitive Dose-Response Curves in Regulatory Networks
Source: PLoS Comput Biol. 2011 Oct 6;7(10):e1002171. doi: 10.1371/journal.pcbi.1002171 (PMC3188500; doi:10.1371/journal.pcbi.1002171)

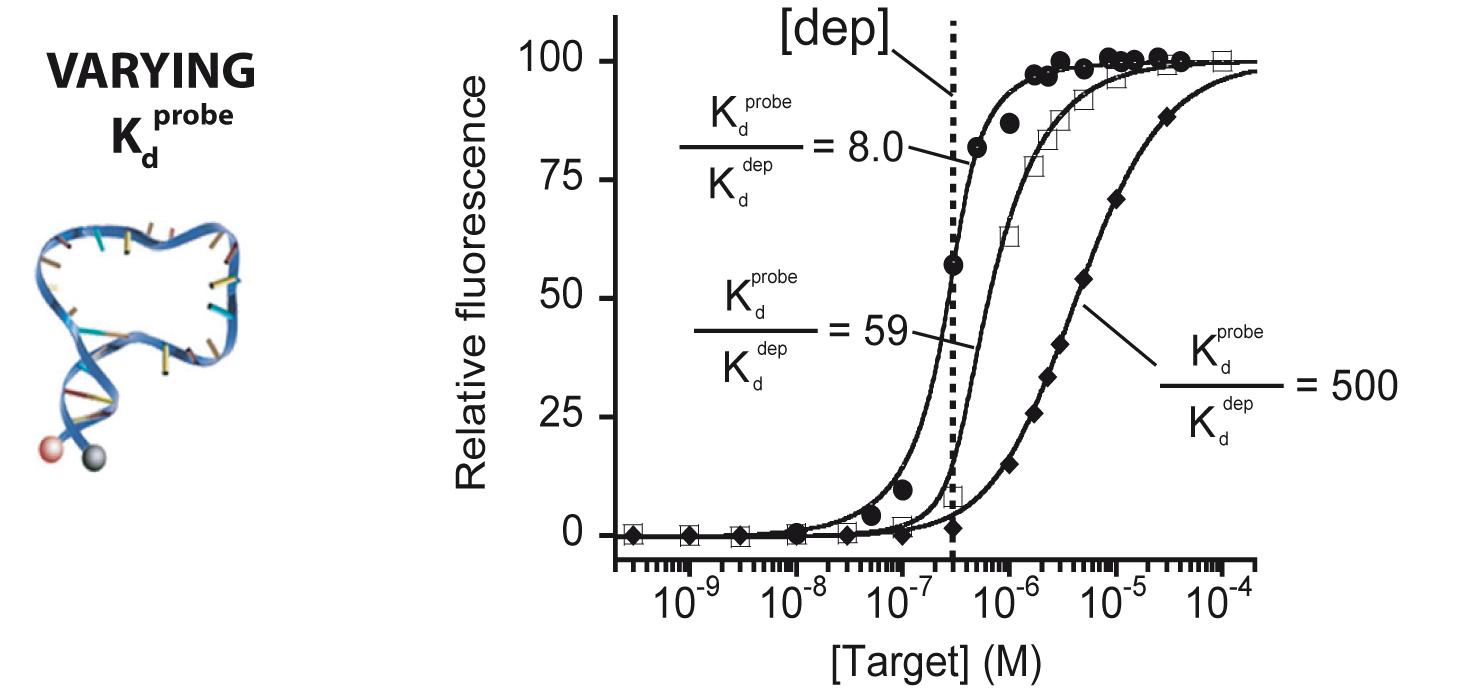

Supplement: Figure S1 — The concentration of the depletant and the affinities of the probe and depletant sometimes interact in complex ways during the generation of ultrasensitivity. Here we demonstrate this by fixing the concentration of the depletant (0GCdep) at 300 nM and employing probes of differing affinities. Despite the high affinities ratio (Kdprobe/Kddep = 500), we do not observe ultrasensitivity when the probe dissociation constant is higher than the concentration of the depletant (3GCprobe; Kdprobe = 2.6 µM, [dep]/Kdprob e = 0.11), because the free target concentration at the “threshold” is too low to saturate the probe. In contrast, we achieve greater sensitivity when the dissociation constant of the probe is close to (2GCprobe; Kdprobe = 310 nM, [dep]/Kdpro be = 0.97) or lower (1GCprobe; Kdprobe = 42 nM, [dep]/Kdprobe = 7.1) than the depletant concentration. The solid lines are estimates taken directly from equation 2 (and using the known dissociation constants of the relevant probes and depletants) without the use of any fitted parameters. (TIF) [file pcbi.1002171.s001.tif]

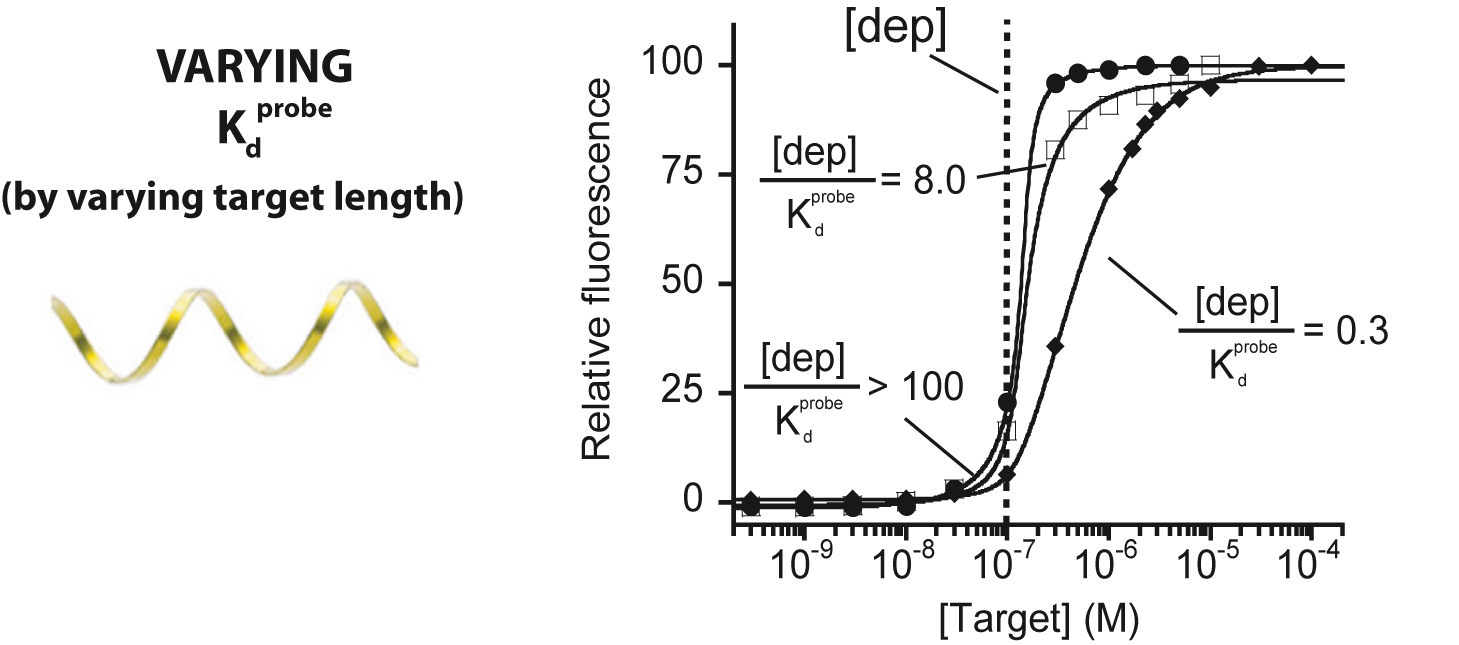

Supplement: Figure S2 — We have also characterized the effects of varying Kdprob e at a constant depletant concentration. We have done this by lengthening the target, which simultaneously improves its affinity for the depletant and for the probe -here 2GC- and keeping the concentration of depletant (here 0GC) constant (i.e. 100 nM). Using targets ranging from 13 to 17 nucleotides (producing lower depletant dissociation constants) we observe a monotonic increase in the pseudo-Hill coefficient from 1.3 to 4.0. This steep, ultrasensitive response is achieved despite the low, 10-fold [depletant]/[probe] ratio employed here, which usually renders the generation of ultrasensitivity more difficult (i.e. [probe] = 10nM). The solid lines are simulations taken directly from equation 2 without the use of any fitted parameters. (TIF) [file pcbi.1002171.s002.tif]
